# Efficacy of Wearable low-intensity pulsed Ultrasound treatment in the Movement disorder in Parkinson’s disease (the SWUMP trial): protocol for a single-site, double-blind, randomized controlled trial

**DOI:** 10.1186/s13063-024-08092-y

**Published:** 2024-04-22

**Authors:** Chuanyu Zhong, Ning Guo, Canfang Hu, Ruilong Ni, Xiaojie Zhang, Zheying Meng, Taotao Liu, Siqi Ding, Wanhai Ding, Yuwu Zhao, Li Cao, Yuanyi Zheng

**Affiliations:** 1 Department of Ultrasound in Medicine, Shanghai Jiao Tong University of Medicine Affiliated Sixth People’s Hospital, Shanghai, 200233 People’s Republic of China; 2 Department of Neurology, Shanghai Jiao Tong University of Medicine Affiliated Sixth People’s Hospital, Shanghai, 200233 People’s Republic of China; 3 Department of Neurology Medical, Jinshan Branch of Shanghai Sixth People’s Hospital, Shanghai, 201599 People’s Republic of China; 4 Department of Neurosurgery, Shanghai Jiao Tong University of Medicine Affiliated Sixth People’s Hospital, Shanghai, 200233 People’s Republic of China

**Keywords:** Parkinson’s disease, Ultrasonic therapy, Neuromodulation, Low-intensity pulsed ultrasound, Randomized controlled trial

## Abstract

**Background:**

Parkinson’s disease (PD) is a progressive, neurodegenerative illness marked by the loss of dopaminergic neurons, causing motor symptoms. Oral levodopa replacement therapy remains the gold standard in the treatment of PD. It is, nevertheless, a symptomatic treatment. There is currently no effective treatment for PD. Therefore, new therapies for PD are highly desirable. Low-intensity pulsed ultrasound (LIPUS) has been shown to improve behavioral functions in PD animal models. It is a new type of neuromodulation approach that combines noninvasiveness with high spatial precision. The purpose of this study is to establish a new clinical protocol for LIPUS in the treatment of movement disorders in patients with PD.

**Methods:**

This protocol is a single-site, prospective, double-blind, randomized controlled trial (RCT). Forty-eight participants with clinically confirmed PD will be randomly allocated to one of two groups: LIPUS group or sham group. All of the participants continue to use pharmacological therapy as a fundamental treatment. The primary outcome is the difference between groups from baseline to 4 months in the change in the Unified Parkinson’s Disease Rating Scale (UPDRS) motor score (part III). The secondary outcomes include the rating scales such as the Mini-Mental State Examination (MMSE), and other three rating scales, and medical examinations including high-density electroencephalography (hdEEG) and functional magnetic resonance imaging (fMRI). The primary safety outcome will be assessed at 4 months, and adverse events will be recorded.

**Discussion:**

This study represents the clinical investigation into the efficacy of therapeutic LIPUS in the treatment of PD for the first time. If LIPUS is determined to be effective, it could offer a practical and innovative means of expanding the accessibility of ultrasound therapy by using a wearable LIPUS device within a home setting.

**Trial registration:**

Chinese Clinical Trial Registry ChiCTR2100052093. Registered on 17 October 2021.

**Supplementary Information:**

The online version contains supplementary material available at 10.1186/s13063-024-08092-y.

## Introduction

### Background and rationale {6a}

Parkinson’s disease (PD) is a progressive, neurodegenerative disorder marked by the dysfunction and loss of multiple neuronal populations, mainly dopamine neurons located in the substantia nigra, which results in the motor symptoms of PD [1]. Movement-related symptoms include bradykinesia, stiffness of the limbs and torso, resting tremor, and postural instability that may be present at the time of diagnosis and deteriorate over time, even causing physical disability [2–4]. 6.1 million people worldwide were diagnosed with PD in 2016, a figure that was 2.4 times greater than in 1990 [4]. The prevalence of PD rises with age, with 1% of the population over 60 years old suffering from the disease [5], which places a heavy financial burden on families and society.

There are numerous treatments available to help with the motor symptoms of PD, including pharmacologic techniques such as levodopa preparations given with or without other drugs [6, 7], as well as non-pharmacologic techniques that include deep brain stimulation (DBS) and non-invasive therapies such as MRI-guided focused ultrasound (FUS) and low-intensity pulsed ultrasound (LIPUS) [8–10]. DBS is typically considered when patients with a diagnosis of PD experience either the “wearing off” phenomenon or dyskinesias, and these experiences do not respond to medication adjustments [1]. However, DBS has some drawbacks with invasiveness, including stimulation tolerance, infection, hemorrhage, and other risks as therapy with implanted devices [9].

With respect to non-invasive ultrasound therapies, an article reported by Science anticipated that it would show promise in the treatment of brain disorders, offering a safe and predictable method for modifying human brain function [11]. It is a new type of neuromodulation technique that combines noninvasiveness with high spatial precision. As a propagating wave, ultrasound can penetrate biological tissues, including the skull, and its energy can be concentrated into a tiny, restricted area, causing a variety of thermal and non-thermal effects on cells and tissues, depending on several parameters such as frequency, intensity, duty cycle, and exposure period on neurons [12, 13]. Recently, a study demonstrated that FUS subthalamotomy in one hemisphere led to improvements in the motor symptoms of PD, marking a significant milestone [14]. Nevertheless, the utilization of MRI-guided FUS to target the thalamus comes with the potential for enduring complications and side effects, such as finger paresthesia, ataxia, and orofacial paresthesia [15]. In addition, FUS ablation at high temperatures may cause thermal lesion of the neural tissues, possibly resulting in irreversible damage [16].

Compared with FUS ablation, LIPUS can be safely used to regulate neuronal circuits in the central nervous system, inducing therapeutic changes without thermal effect [17, 18]. LIPUS allows for non-invasive penetration through the skull with neuroprotective and reversible neuromodulatory effects, adjustability, and high spatial resolution [19, 20]. Furthermore, pulsed ultrasound is an intermittently delivered ultrasound that minimizes the thermal effects of ultrasound and targets the tissue cells of the lesion without affecting the normal tissue cells [21]. In numerous PD animal models, LIPUS has been shown to stimulate or inhibit neuronal activity and stimulate the motor cortex [22–24]. These findings indicate that LIPUS holds promise as a modality for neuromodulation. Furthermore, to the best of our knowledge, no clinical trial has previously employed LIPUS treatment in patients with PD.

Therefore, the current single-site, prospective, double-blind, randomized controlled trial (RCT) aims to look into the efficacy of LIPUS in the treatment of PD. The trial is the first clinical trial using the self-developed wearable LIPUS device for PD treatment.

### Objectives {7}

The aim of the present study is to evaluate the efficacy of LIPUS as a therapeutic intervention for alleviating motor impairments in individuals with PD. In order to achieve the aim of this study, the subsequent research inquiries have been formulated:

1. Is the ultrasound stimulation administered in the LIPUS group more effective than the sham group without ultrasound stimulation in treating movement disorders in PD patients?
2. What cerebral modifications do patients experience as a result of LIPUS therapy?
3. Apart from motor symptoms, are there changes in other symptoms, like depression and anxiety, that may occur?

### Trial design {8}

**Fig. 1:**
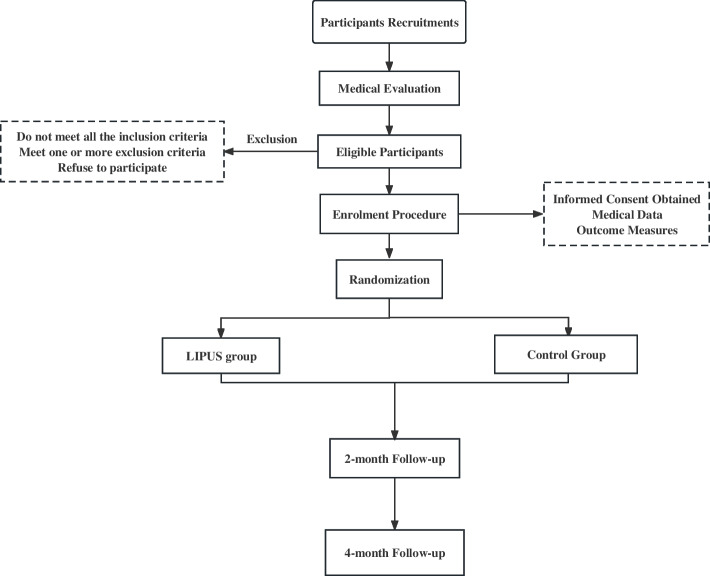
Participant flow chart

The trial is a prospective, double-blind, single-site control clinical superiority trial that will enroll patients diagnosed with PD based on the U.K. Brain Bank Clinical Criteria from Shanghai Jiao Tong University of Medicine Affiliated Sixth People's Hospital [25]. Participants will be randomly assigned to two groups in a 1:1 allocation ratio: the LIPUS group (600 kHz, 1.0 W/cm^2^) and the sham group. This manuscript follows the Standard Protocol Items: Recommendations for Interventional Trials (SPIRIT) guideline [26]. The flow chart depicting the study protocol is presented in Fig. 1.

## Methods: participants, interventions, and outcomes

### Study setting {9}

This is a single-center, prospective, double-blind, randomized controlled trial that will be conducted at the Shanghai Jiao Tong University of Medicine Affiliated Sixth People’s Hospital.

### Eligibility criteria {10}

Inclusion criteria are as follows: (1) subjects with a diagnosis of PD based on UK Brain Bank Criteria as confirmed by a neurologist; (2) Hoehn and Yahr stage in the on-medication state of 2.5 or less; (3) in principle, the pharmacological regimen remains unchanged during LIPUS treatment; (4) men and women, ranging in age from 18 to 80 years old; (5) available to participate in follow-up for the duration of study and give consent; (6) subjects are examined by transcranial sonography, having a sufficient temporal bone window and substantia nigra echogenicity (contralateral and ipsilateral) so that they can be targeted by a wearable LIPUS device.

Exclusion criteria are as follows: (1) Hoehn and Yahr stage of 2.5 or higher; (2) presence of another central neurodegenerative disease. These include Parkinsonism-Plus syndrome, dementia with Lewy bodies, Alzheimer’s disease, and amyotrophic lateral sclerosis; (3) previous history of intracranial hemorrhage or severe head injury; (4) a history of ischemic stroke or a stroke within the previous 6 months; (5) subjects with malignant brain tumors or a history of seizures within the past year; (6) pregnancy or lactation; (7) Parkinsonian symptoms, which are a side effect of any medication; (8) subjects who have previously undergone deep brain stimulation or nerve nucleus ablation; (9) subjects exhibiting any ethanol or substance abuse behaviors; (10) current medical condition that is causing abnormal bleeding and/or coagulopathy; (11) subjects who are unable to communicate with the researchers or participate in other clinical studies in the meantime.

### Who will take informed consent? {26a}

The research assistant (C. Z.) will obtain informed consent from participants before conducting the baseline evaluation in the participant reception room. Participants will engage in a 30-min group discussion with a research assistant. This session will encompass an overview of the PD therapy protocol for the trial and detailed instructions on how to use the wearable LIPUS device. Participants will receive an explanation of the study’s purpose, their role in it, and the potential benefits and risks involved. Meanwhile, participants will also be provided with an instructional video, produced by the research team, demonstrating the correct usage of the LIPUS device. If participants have any questions, they are encouraged to ask, and they have the freedom to decide whether to participate in the study after fully comprehending the information provided.

### Additional consent provisions for collection and use of participant data and biological specimens {26b}

No additional biological samples were collected during the study, and no subject data are currently available for future research endeavors. If any participant data relevant to subsequent studies are required, additional consent will be obtained from the participants.

### Interventions

#### Explanation for the choice of comparators {6b}

To evaluate the efficacy of LIPUS therapy, we chose the use of null stimulation as the comparator. It is important to note that each LIPUS wearable device is equipped with mechanical vibration, whether or not ultrasonic waves are employed. The subjects are unable to distinguish the presence or absence of ultrasonic stimulation.

### Intervention description {11a}

Participants will self-administer LIPUS treatment at home for 4 months using a self-developed wearable LIPUS device based on dopaminergic medication, the quantity of which will not be adjusted during the trial until the condition worsens or adverse effects occur. The LIPUS group received pulsed therapeutic ultrasound (Shanghai, China) through the temporal bone window with a frequency of 600 kHz, an intensity of 1.0 W/cm^2^, a duty cycle of 50% (10 ms on, 10 ms off), and a duration of 30 min. This LIPUS treatment will be conducted once a day for a total duration of 4 months.

Each participant will be initially guided by ultrasound through the skull to the bilateral substantia nigra and striatum by a senior radiologist [27]. Then, the radiologist will mark the stimulation site with a marker, and a research assistant will capture a photograph to document the precise targeted site for each participant. Typically, the targeted site is usually at the tip of the ear [27, 28]. The treatment site of the LIPUS device will be positioned on these two marked target sites.

Participants in the sham group, who will only receive the basic medication, will also be provided with ultrasound devices that do not have any therapeutic effect without LIPUS stimulation. The trial team’s other procedures and activities will be consistent and identical for both groups.

### Criteria for discontinuing or modifying allocated interventions {11b}

In this study, the criteria for discontinuing the intervention will be (1) participants who are unable to complete the clinical study, (2) participants who voluntarily withdrew informed consent, and (3) participants who experience serious adverse events. As of now, no adverse events have been reported during the course of LIPUS treatment.

### Strategies to improve adherence to interventions {11c}

To enhance compliance with the interventions, we offer trial-related assessments of the rating scales and medical examinations, which encompass ultrasound examinations, high-density electroencephalography (hdEEG), and functional magnetic resonance imaging (fMRI) from the baseline to the 4-month treatment period at no cost to the participants.

### Relevant concomitant care permitted or prohibited during the trial {11d}

All participants will be provided with a pharmacological regimen as part of the trial. It is strongly discouraged for participants to seek additional treatments beyond what is assigned in the protocol during the intervention period. Participants are obligated to report any deviations from the protocol, which includes adjusting medications for PD or adding other treatments.

### Provisions for post-trial care {30}

Trial interventions are designed to inflict minimal to no harm. If a participant suffers any injury due to participating in the trial, we will evaluate, document, provide appropriate medical care, and pay for all related medical expenses.

### Outcomes {12}

#### Primary outcome

The primary effectiveness outcome will be the difference in mean change (at baseline, 2 months, and 4 months) across groups (LIPUS vs sham treatment) according to blinded site evaluation of the UPDRS III score for the affected side. The primary outcome is calculated as the sum of the unilateral items for the following: speech, facies, tremor in resting, intentional tremor, rigidity, rapid movements of the fingers, rapid hand movements, alternating movements, leg movements, getting up from a chair, posture, posture stability, starting walking, and bradykinesia in the third section (motor). The scoring system includes the following: 0 points = no involvement; 1 = detectable disorders; 2 = moderate disorders; 3 = considerable disorders; 4 = no function or severe disorders [29]. Therefore, the primary endpoint score ranges from 0 to 56, with higher scores suggesting worse parkinsonism.

Meanwhile, Fahn and Elton created the Unified Parkinson’s Disease Rating Scale (UPDRS), which is made up of six elements. These are as follows: (I) the state of intellectual and mood disorders; (II) activities of daily living (separately for phase “on” and “off”); (III) motor examination; (IV) complications of treatment; (V) stages of the disease; (VI) self-assessment of independence using the Schwab and England Scale. Furthermore, the subjective, patient-derived Activities of Daily Living (Part II) component of the UPDRS corresponds well with the objective, physician-derived motor section (Part III) [30].

### Secondary outcomes

The following secondary outcomes of this trial will be measured during the 4-month follow-up visit as listed and explained below:

The MMSE (Mini-Mental State Exam) for assessment of cognitive function (includes tests of orientation, attention, memory, language, and visual-spatial skills; range 0–30 with a higher score suggesting better cognitive function) at baseline, 2 months, and 4 months [31].

The BDI (Beck Depression Inventory) for measurement of the severity of depression (range 0–63 with a higher score indicating more severe depressive symptoms) at baseline, 2 months, and 4 months [32].

The BAI (Beck Anxiety Inventory) for measurement of anxiety (range 0–63 with a higher score indicating more severe anxiety symptoms) at baseline, 2 months, and 4 months [33].

High-density electroencephalography (hdEEG) for the assessment of cognitive function changes at baseline, 2 months, and 4 months [34].

Functional magnetic resonance imaging (fMRI) to investigate brain changes associated with the motor and non-motor symptoms at baseline and 4 months [35].

### Participant timeline {13}

The participant timeline is shown in Table 1.

**Table 1:** Study evaluation procedures and timeline

Study procedure	Medical evaluation	Enrolment visit	2 months	4 months
Determine eligibility	√	√		
Obtain signed consent		√		
Other medical and demographic data		√		
Give instructions for LIPUS devices		√		
Outcome measures				
Unified Parkinson’s Disease Rating Scale		√	√	√
Other rating scales		√	√	√
High-density electroencephalography		√	√	√
Functional magnetic resonance imaging		√		√

#### Sample size and power {14}

The sample size and power calculations were centered on the primary outcomes, which pertained to the alterations in UPDRS III scores from the baseline assessment to the 4-month follow-up assessment after 4 months of LIPUS treatment. In accordance with the findings derived from the limited sample study involving 20 patients, the LIPUS group exhibited a mean change of 5.54 ± 8.03, while the sham group showed a mean change of − 0.71 ± 2.25. The researcher utilized the sample size calculation software PASS to compute the sample size for each group, which was *n*_1_ = *n*_2_ = 21 (1:1 allocation ratio), to detect differences with a two-sided significance level of 0.05 and 90% power calculations. With the consideration of a 10% dropout rate, the final sample size is expected to be 48. In the prior investigation [36], wherein a clinically meaningful alteration in UPDRS III scores was established as a 5-point disparity, the study determined that the efficiency of the existing sample size in power calculations reached 0.8078.

#### Recruitment {15}

Figure 1 shows the participant flow chart throughout the trial. Social media will be used in the recruitment of participants, which is planned to take 18 months, beginning in May 2022. Recruitment will also be carried out at Shanghai Sixth People’s Hospital, Shanghai Jiao Tong University, Shanghai, China. For those who are interested in the trial, a face-to-face interview will be scheduled by an experienced researcher. The subjects and their guardians will be informed about the study’s purpose, techniques, advantages, and any discomfort or risks. If they understand the information, they will sign the informed consent form.

## Assignment of interventions: allocation

### Sequence generation {16a}

Participants will undergo randomization, being assigned to either the LIPUS group or the sham group in a 1:1 ratio, facilitated by a computer-generated randomization sequence. To safeguard the impartiality and autonomy of the allocation data, research assistants who are not engaged in outcome assessment will be responsible for generating sequentially numbered opaque sealed envelopes according to the randomization list.

### Concealment mechanism {16b}

These envelopes will be used to maintain the confidentiality and independence of the allocation process. When necessary, the designated assistant will unseal the envelopes and oversee the coordination of therapeutic interventions.

### Implementation {16c}

The sealed envelopes will be opened and resealed during the initial stage by the staff in charge of randomization. Subsequently, the code will be communicated to the LIPUS experimenter.

## Assignment of interventions: blinding

### Who will be blinded {17a}

Both patients and clinic staff members, including caregivers, outcome assessors, and statistical analysts, will be kept unaware of the group assignments. The distinction between whether the intervention involves LIPUS or null stimulation will not be disclosed at any point during the study. Clinicians Y.Z., L.C., and Y.Z. have access to the final trial dataset.

### Procedure for unblinding if needed {17b}

After the completion of the analysis of primary and secondary outcomes, the identities of the two groups will be unveiled.

## Data collection and management

### Plans for assessment and collection of outcomes {18a}

The primary outcome data will be evaluated at baseline, after 2 months of treatment, and after 4 months of treatment. Secondary outcomes, which encompass additional rating scales and hdEEG, will likewise be assessed at baseline, after 2 months of treatment, and after 4 months of treatment. Furthermore, secondary outcomes, such as fMRI, will be acquired at the baseline and upon completion of the treatment. All variables outlined in the protocol will be meticulously recorded within an encrypted Microsoft Office Excel document. The investigators responsible for data entry in this Office Excel file hold the duty of upholding data accuracy and completeness. To supervise and administer the trial data, a management team has been established, composed of the principal investigator, senior investigator, a research assistant, and a doctorate student each with their respective responsibilities for tasks.

### Plans to promote participant retention and complete follow-up {18b}

Patients will be required to participate in all evaluations and treatments over the course of the 4-month trial. The patients will be provided with trial-related assessments, such as rating scales and medical examinations, which will include ultrasound examinations, hdEEG, and fMRI, without incurring any financial expenses or burdens.

If a participant chooses to discontinue their involvement in the study during the therapy phase and fails to complete the entire 4-month treatment duration, their data will not be maintained for the purpose of analysis. It is imperative to exert all conceivable efforts in order to incentivize and provide assistance to patients, ensuring their sustained participation in the study until the completion of their intended outcome assessments.

### Data management {19}

Clinical data will be collected and managed using an electronic database during participants’ hospital visits at baseline, 2 months, and 4 months (Table 1). The paper version materials, which include the protocol, case reported forms, informed consent forms, and electronic version database, will be kept in an independently secured box at Shanghai Sixth People’s Hospital by the principal investigator. The research assistants will send out reminder emails and make phone calls to ensure that participants complete the research. If (1) the participant withdraws his or her consent and (2) exclusion criteria are discovered after registration, the participant will be removed from the study. The reason for the suspension as well as the date of the suspension will be documented. The consent form will include permission to utilize data collected prior to the participant’s withdrawal.

### Confidentiality {27}

All personal data of registered participants will be assigned a unique identifier and stored on a secure server available only to researchers with administrative privileges. After the trial, only non-personal data will be available for analysis in the data repository.

## Plans for collection, laboratory evaluation, and storage of biological specimens for genetic or molecular analysis in this trial/future use {33}

Not applicable; no biological specimens will be collected.

## Statistical methods

### Statistical methods for primary and secondary outcomes {20a}

SPSS 26.0 will be used for the statistical analysis. Continuous variables will be represented by a mean and standard deviation, whereas categorical variables will be represented by counts and percentages. The intention-to-treat principle will be used to analyze the UPDRS III score (primary outcome) [37], which will include data from all randomized individuals. A per-protocol analysis of other outcomes from the participants who completed the entire research will also be performed. Multiple imputations will be used to replace missing data using multivariable regression models with chained equations. The mean treatment differences and 95% CIs between measures with LIPUS and sham-LIPUS will be computed. All statistical tests will be two-sided with a *p* < 0.05 statistical significance level.

### Interim analyses {21b}

No interim analyses will be planned.

### Methods for additional analyses (e.g., subgroup analyses) {20b}

No subgroup analysis will be conducted.

### Methods in analysis to handle protocol non-adherence and any statistical methods to handle missing data {20c}

To mitigate protocol non-adherence, participant education will place significant emphasis on the importance of adhering to the protocol. Meanwhile, investigators and research staff will receive comprehensive training and ongoing support. Furthermore, a WeChat group will be established for each participant. Within this group, daily treatment photos will be shared to facilitate supervision and identification of stimulation sites.

We anticipate minimal missing data in the outcome (rating scale) assessments, as we have provisions in place to assess participants remotely using a dedicated mobile application in the event that they are unable to visit the hospital during the treatment phase. Missing data will be imputed using the last observation carried forward (LOCF) analysis.

### Plans to give access to the full protocol, participant-level data, and statistical code {31c}

Anonymized participant data may be provided by the corresponding author upon reasonable request. However, it should be noted that there are no plans to grant public access to participant data and the statistical code.

## Oversight and monitoring

### Composition of the coordinating center and trial steering committee {5d}

The management group responsible for conducting clinical monitoring activities will consist of several members, including the principal investigator, senior investigator, a research assistant, and a doctorate student, which will meet on a monthly frequency. It is important to highlight that the present study does not incorporate a trial steering group.

### Composition of the data monitoring committee, its role, and reporting structure {21a}

This trial does not have a formal data monitoring committee in place, as there are no interim analyses planned, and there are no procedures for early stopping.

### Adverse events reporting and harms {22}

From the start of the trial (either LIPUS or sham treatment) to the 4-month follow-up visit, the primary safety endpoint is the occurrence of adverse events. The primary outcome is a four-month safety visit. Meanwhile, research visits will be scheduled every two months. Adverse events will be assessed using the following criteria: those reported spontaneously by participants, those reported in response to a particular inquiry for adverse events, and those noticed by the neurologists during the general and neurological examinations at each visit. All adverse events that occur throughout the trial will be meticulously documented.

### Frequency and plans for auditing trial conduct {23}

Not applicable. There are no auditing plans for this trial. The researchers will conduct weekly meetings throughout the study period to review and monitor the trial’s progress.

### Plans for communicating important protocol amendments to relevant parties (e.g., trial participants, ethical committees) {25}

Any modifications to the protocol must receive prior approval from the Ethics Committee of Shanghai Sixth People’s Hospital. If these modifications are approved, they will be duly reported in the trial register and incorporated into the final research data report.

## Dissemination plans {31a}

The results of this clinical trial are anticipated to be disseminated through publication in scientific medical journals as well as the presentation at national and international conferences. The first and corresponding author will assume the primary responsibility for the publication of these findings. Outcome summaries will be disseminated to both participants and clinical staff involved in the trial.

## Discussion

The degeneration of cells in the substantia nigra and the subsequent reduction in dopamine synthesis within the basal ganglia are the primary causes of movement disorders in PD. Under typical physiological circumstances, the substantia nigra and striatum play a crucial role in regulating movement [38]. At present, the primary strategy for managing PD is the administration of symptomatic drugs that aim to either increase dopamine levels or directly stimulate dopamine receptors.

For non-invasive ultrasound therapies, high-intensity FUS has predominantly been used in the treatment of PD patients who present with tremor as their predominant symptom, which has received approval from the Food and Drug Administration [15]. With the guidance of MRI, high-intensity FUS is employed to effectively enter the brain and establish stable focal points within the intracranial region. The absorption of ultrasound occurs inside the specific region of interest, resulting in the production of thermal energy. This results in elevated temperatures at the focal point due to the thermal, mechanical, and cavitation effects, leading to protein denaturation, coagulation, and cell necrosis within the targeted tissues, ultimately leading to thermal ablation. The findings of several studies indicate that the implementation of unilateral FUS subthalamotomy and pallidotomy may potentially result in an improvement in the motor symptoms commonly observed in individuals diagnosed with PD [14, 39, 40]. Nonetheless, the follow-up results revealed that certain patients had adverse reactions including dyskinesia, gait disturbances, loss of taste, and facial weakness [39–41]. At the same time, the effectiveness of LIPUS in improving movement disorders in animal models of PD has been established. LIPUS is a type of ultrasound that operates in a pulsed wave mode and is typically delivered at significantly lower intensity levels, usually below 3 W/cm^2^, which is in accordance with safety standards and clinical practice. Also, studies have shown that low-frequency ultrasound, typically at frequencies less than 0.7 MHz, can be effectively transmitted through the skull [42, 43].

Hence, our research opts for a LIPUS treatment regimen consisting of a frequency of 600 kHz, an intensity of 1.0 W/cm^2^, and a daily duration of 30 min. This therapy protocol will be implemented over a period of 4 months to investigate its potential therapeutic impact on the motor symptoms experienced by individuals with PD. Meanwhile, our team has developed a wearable LIPUS device that allows patients to undergo treatment in their homes, eliminating the need to visit the hospital daily and providing time-saving convenience.

This study marks the pioneering clinical trial involving LIPUS in the treatment of patients with PD. LIPUS exhibits substantial promise as a neuromodulation modality. It has the ability to penetrate the skull and impart neuroprotective and reversible neuromodulatory effects. The capacity for reversible neuromodulation through LIPUS presents a new opportunity for the treatment of individuals in the early stage of PD.

Finally, this study has certain limitations. On the one hand, this trial is a single-site RCT involving a relatively small number of individuals with PD. In our next phase, we intend to undertake a multi-center clinical study to broaden the pool of participants and enhance the generalizability of our findings. On the other hand, involvement in the study necessitates the inclusion of individuals with a favorable temporal window for ultrasound penetration. This selection criteria may, unfortunately, exclude individuals who are unable to receive ultrasound through the temporal window, thus preventing them from accessing LIPUS treatment.

## Trial status

The initial version of the protocol received approval from the ethical committee in February 2022, and this protocol is designated as version 3.0. Patient recruitment and data collection will take place at our hospital from May 2022 to November 2023, with subsequent data compilation and analysis. The expected last visit date is February 2024. As a result of prior submissions to other journals, the manuscript underwent a peer review process that lasted over a year, ultimately resulting in rejection. Consequently, an earlier submission was unattainable.

### Supplementary Information

**Supplementary Material 1.**

## Data Availability

The datasets produced and examined in this work are not accessible to the public due to the ongoing completion of the protocol at the time of submission. However, interested parties may obtain access to these datasets by contacting the corresponding author and making a fair request.

## Abbreviations

LIPUS: Low-intensity pulsed ultrasound
PD: Parkinson’s disease
RCT: Randomized controlled trial
UPDRS: Unified Parkinson’s Disease Rating Scale
MMSE: Mini-Mental State Examination
BDI: Beck Depression Inventory
BAI: Beck Anxiety Inventory
hdEEG: High-density electroencephalography
fMRI: Functional magnetic resonance imaging
FUS: Focused ultrasound
LOCF: Last observation carried forward
