# Supplementary material for: Efficacy of Wearable low-intensity pulsed Ultrasound treatment in the Movement disorder in Parkinson’s disease (the SWUMP trial): protocol for a single-site, double-blind, randomized controlled trial
Source: Trials. 2024 Apr 22;25:275. doi: 10.1186/s13063-024-08092-y (PMC11036625; doi:10.1186/s13063-024-08092-y)
Supplement: Supplementary file 1 — Supplementary Material 1. [file 13063_2024_8092_MOESM1_ESM.zip › SWUMP Ethics Approval Document-English version.pdf]

The Ethics Committee of the
Sixth People's Hospital of
Shanghai Municipality

Approval by the Ethics Committee of Shanghai Sixth People's Hospital

Approval Letter of Ethics Committee of Shanghai Sixth People's Hospital

Approval No. Approval No:2022-023 Review Date Date of Review: 2022-02-24

Project name: Study Title	Clinical study of wearable low-intensity ultrasound for movement disorders in patients with Parkinson's disease			Type of application Application Type	Clinical research
Drug name Drug Name	/	Drug category Drug Category	/	clinical stages Phase of drug study	/

Clinical trial approval number and issuing unit (Clinical Trial Approva l No.and Issued By): CFDA 20200121	Drug test report and batch number (Certificate of Anal ysis and Batch No.):
Principal Investigator (Principal Investigator): Medical Imaging (Ultrasound Department) Zheng Yuanyi	The Sponsor Unit (Sponsor): Shanghai Sixth People's Hospital / CRO: None

The following study-related documents have been reviewed:
The following items have been reviewed in connection with the above study to be conducted by the above Investigato r

☑ Study protocol and date Protocol No.and dated: Version Number: 2.0; Version Date: 20220212

☑ Patient Informed Consent Form and Date Consent From (s) dated: Version Number: 3.0; Version Date: 20220218

☐ Protocol modification and date Protocol Amendment (s) dated:

☑ Patient Recruitment Advertising and Date Advertisements for Recruitment dated: Version Number: 2.0; Version Date: 20220210

☑ Other (please list) Other (specify): ethical review application form; list of documents submitted for review; description of subject recruitment method; clear document of clinical study registration certificate

Document; resume of principal researchers of the center; GCP certificate (1.0,20220218); pre-evaluation opinions of product technical requirements

(1.0,20220218); self-inspection report (1.0,20220221); the production of medical devices meets the requirements of the medical device quality management system

(1.0,20220221); Product Registration and Test Report (1.0,20220218)

Vote result Voting

On February 24,2022, the Ethics Committee carefully discussed the submitted materials and voted. 12 participants attended the meeting, 12 participants voted for 0 votes, 12 votes agreed, 0 votes for necessary modification (quick review), 0 votes for necessary modification (review by meeting), and 0 votes disagreed.

Review decision Decision: (in oral)

☑ Consent with Approval: After the review of the Ethics Committee, we agreed to perform the study according to the approved clinical study protocol, informed consent form, and recruitment materials.

Annual / follow-up review frequency	Every 12 months from the date of approval of the study (Ethics changes changes on actual progress Right to continuous review frequency.)
Validity of approval:	In 1 year
Signature of Ethics Chairman / Deputy Chairman Signature Chair / Deputy of [illegible] Ethics Committee: Hospit[illegible]l Ethics Committee (seal)[illegible] date: [illegible]	
statement Statements<br>1. The relevant work of the Ethics Committee of Shanghai Sixth People[illegible]s Hosp[illegible]l fo[illegible]s the Drug Clinical Quality Management Regulations issued by the Food and Drug Admini[illegible]stratio[illegible] [illegible] the People's Republic of China<br>Fan (GCP), Guiding Principles for ethical Review of Drug Clinical Trials and ethical review principles of ICH-GCP comply with the requirements of the Declaration of Helsinki and comply with China<br>Provisions of the relevant laws and regulations.	

2. Please follow the GCP principles and the protocol approved by the ethics committee to protect the health and rights of the subjects

3. If the principal investigator is changed during the study, any modification of the clinical study protocol, informed consent form, recruitment materials, etc., shall be reviewed and approved by the Ethics Committee

To implement.

4. Serious adverse events or unexpected adverse events of the center should be notified to the EC pending a new decision.

5. The approval letter is valid for 1 year, and the projects that are not started within the validity period shall be re-reviewed; the investigator shall submit the progress report 1 month prior to the expiration date of the approval letter to continue the study

For the project of the bed test team leader unit, the sponsor shall submit a summary report of the research progress of each center to the ethics committee.

6. In case of any situation that may significantly affect the conduct of the trial or increase the risk of the subject, the applicant shall timely submit a written report to the Ethics Committee.

7. The inclusion of subjects who did not meet the inclusion criteria or meet the exclusion criteria, the subjects from the study, have not followed the protocol; or may not contribute to the rights / health of the subjects and the scientific nature of the study

In case of violation of GCP principle, ask the sponsor / monitor / investigator to submit the protocol violation report.

8. If the applicant suspends or terminates the clinical study early, please submit the suspension / termination study report in time.

9. To complete the clinical study, please submit the final report.

10. The application type that is not drug clinical trial / medical device clinical trial shall not be used for product clinical registration application.

11. Please register this clinical study on the website of China Clinical Trials Registry before recruiting the first subject (website address:http://www.chictr.org.cn/),

And inform the ethics committee of the registration number after registration.

12. International cooperation projects involving human genetic resources and materials (including Sino-foreign cooperation projects implemented in China) shall be reviewed by the China Human Genetic Resources Management Office

It can be officially started only after the approval.

13. Clinical trials of products listed in the category III medical devices to be approved for clinical trial approval shall only be examined and approved by the China Food and Drug Administration

officially launch.

The Ethics Committee of the Sixth People's Hospital of Shanghai Municipality

List of members of the Ethics Committee

ETHICS COMMITTEE COMPOSITION

Approval No. Approval No:2022-023 Approval date Date of Review: 2022 [illegible]-2

Name and professional titles of the committee members<br>Member<br>Name and Title		occupation<br>Occupation (position)	sex<br>Male/Female	work unit<br>Working Place
surname and personal name	professional ranks and titles			
Jia Weiping<br>Jia Wei Ping	professor<br>Professor	doctor<br>Doctor	woman<br>female	Shanghai Sixth People's Hospital<br>Shanghai Sixth People 's Hospita
Zeng Bingfang<br>Zeng Bing Fang	professor<br>Professor	doctor<br>Doctor	man<br>male	Shanghai Sixth People's Hospital<br>Shanghai Sixth People's Hospital
Guo Cheng<br>Guo Cheng	professor<br>Professor	a senior pharmacist<br>Pharmacist	man<br>male	Shanghai Sixth People's Hospital<br>Shanghai Sixth People's Hospital
Bao Yuqian<br>Bao Yu Qian	professor<br>Professor	doctor<br>Doctor	woman<br>female	Shanghai Sixth People's Hospital<br>Shanghai Sixth People's Hospital
Zhou Hongling<br>Zhou Hong Ling	boffin<br>Researcher	Researcher<br>Researcher	woman<br>female	The Shanghai Institute of Space Advancement<br>Sh anghai Institute of Spacecraft Propulsio n
Shen Chengliang<br>Shen Cheng Liang	lawyer<br>Lawyer	lawyer<br>Lawyer	man<br>male	Shanghai Kangzheng Law Firm<br>Shanghai Kangzheng Law Firm
Yan Zhongcheng<br>Yan Cheng Zhong	professor<br>Professor	teacher<br>Teacher	man<br>male	Donghua University<br>DongHua University
Hu Cheng<br>Hu Cheng	boffin<br>Researcher	Researcher<br>Researcher	man<br>male	Shanghai Sixth People's Hospital<br>Shanghai Sixth People 's Hospita
Shen Zan<br>Shen Zan	professor<br>Professor	doctor<br>Doctor	man<br>male	Shanghai Sixth People's Hospital<br>Shanghai Sixth People 's Hospita
Ren Tao<br>Ren Tao	professor<br>Professor	doctor<br>Doctor	man<br>male	Shanghai Sixth People's Hospital<br>Shanghai Sixth People 's Hospita
Shen Chengxing<br>Shen Cheng Xing	professor<br>Professor	doctor<br>Doctor	man<br>male	Shanghai Sixth People's Hospital<br>Shanghai Sixth People's Hospital
Shi Wenjing<br>Shi Wen Jing	professor<br>Professor	doctor<br>Doctor	woman<br>female	Shanghai Sixth People's Hospital<br>Shanghai Sixth People's Hospital
