# Supplementary material for: Efficacy of Wearable low-intensity pulsed Ultrasound treatment in the Movement disorder in Parkinson’s disease (the SWUMP trial): protocol for a single-site, double-blind, randomized controlled trial
Source: Trials. 2024 Apr 22;25:275. doi: 10.1186/s13063-024-08092-y (PMC11036625; doi:10.1186/s13063-024-08092-y)
Supplement: Supplementary file 1 — Supplementary Material 1. [file 13063_2024_8092_MOESM1_ESM.zip › SWUMP Ethics Approval Document-original version.pdf]

上海市第六人民医院伦理委员会审查批件
Approval Letter of Ethics Committee of Shanghai Sixth People's Hospital

审批编号 Approval No：2022-023　　审阅日期 Date of Review：2022-02-24

项目名称 Study Title	可穿戴低强度超声治疗帕金森病患者运动障碍的临床研究			申请类型 Application Type	临床科研
药物名称 Drug Name	/	药物类别 Drug Category	/	临床分期 Phase of drug study	/

临床试验批文号及发文单位 (Clinical Trial Approval No. and Issued By): CFDA 20200121	药检报告及批号 (Certificate of Analysis and Batch No.)： /
主要研究者（Principal Investigator）：医学影像（超声科） 郑元义	申办单位（Sponsor）：上海市第六人民医院/CRO:无

下面的研究相关文件已经审阅:
The following items have been reviewed in connection with the above study to be conducted by the above Investigator:

☑研究方案及日期　Protocol No. and dated：版本号：2.0;版本日期：20220212

☑病人知情同意书及日期 Consent From(s) dated:版本号:版本号：3.0;版本日期：20220218

☐方案修改及日期 Protocol Amendment(s) dated：

☑病人招募广告及日期 Advertisements for Recruitment dated：版本号：2.0;版本日期：20220210

☑其他（请具体列出）Other(specify)：伦理审查申请表;送审文件清单;受试者招募方式说明;临床研究注册证明文件;项目任务书;本中心主要研究者简历、参加研究人员名单;GCP证书(1.0,20220218);产品技术要求预评价意见(1.0,20220218);自检报告(1.0,20220221);医疗器械生产符合医疗器械质量管理体系相关要求的说明(1.0,20220221);产品注册检测报告(1.0,20220218)

投票结果Voting:
伦理委员会于2022年02月24日 对报送材料进行了认真的讨论并进行了投票表决，参加会议人数12 人，回避0人，投票人数12人，12票同意，0票必要的修改后同意（快速审查），0票必要的修改后同意（会议审查），0票不同意。

审查决定*Decision*：（在☐内划×）

☑同意 Approval ：经本伦理委员会审查，同意按所批准的临床研究方案、知情同意书、招募材料开展本项研究。

年度/跟踪审查频率：	为该研究批准之日起每12个月一次（伦理委员会有根据实际进展情况改变持续审查频率的权利。）
批件有效期：	1年

伦理委员会主任委员/副主任委员签名Signature Chair/Deputy of Ethics Committee：
医院伦理委员会（盖章）：
日期：2022/0313

声明Statements：

1、上海市第六人民医院伦理委员会的相关工作遵循中华人民共和国食品药品监督管理总局颁发的《药物临床质量管理规范(GCP)》、《药物临床试验伦理审查工作指导原则》和ICH-GCP的伦理审查原则，符合赫尔辛基宣言的要求，并遵守中国相关法律和法规的规定。

地址：上海市宜山路600号　邮编：200233　电话：64369181　传真：64368920

2、请遵循GCP原则、遵循伦理委员会批准的方案开展临床研究，保护受试者的健康与权利。
3、研究过程中若变更主要研究者，对临床研究方案、知情同意书、招募材料等的任何修改，需经伦理委员会审查同意后方可实施。
4、本中心发生的严重不良事件或非预期不良事件需向本伦理委员会通报，并等待做出新的决定。
5、批件有效期为1年，在有效期内未启动的项目需要重新进行审查；研究者须在批件有效期（若规定的年度/定期跟踪审查频率短于批件有效期，须在规定的持续审查日期）到期前1个月递交进展报告，以获得批准持续进行研究；本院作为临床试验组长单位的项目，申办者应当向伦理委员会提交各中心研究进展的汇总报告。
6、当出现任何可能显著影响试验进行、或增加受试者危险的情况时，请申请人及时向伦理委员会提交书面报告。
7、研究纳入了不符合纳入标准或符合排除标准的受试者，符合中止试验规定而未让受试者退出研究，给予错误治疗或剂量，给予方案禁止的合并用药等没有遵从方案开展研究的情况；或可能对受试者的权益/健康、以及研究的科学性造成不良影响等违背GCP原则的情况，请申办者/监查员/研究者提交违背方案报告。
8、申请人暂停或提前终止临床研究，请及时提交暂停/终止研究报告。
9、完成临床研究，请申请人提交结题报告。
10、申请类型不是药物临床试验/医疗器械临床试验的批件不得用于产品临床注册申请。
11、请于募集第一名受试者之前在中国临床试验注册中心网站注册此项临床研究（网站地址：http://www.chictr.org.cn/），并在注册后将注册号告知伦理委员会。
12、涉及人类遗传资源材料的国际合作项目（包括在境内执行的中外合作项目），需经中国人类遗传资源管理办公室审核批准后方可正式启动。
13、列入需进行临床试验审批的第三类医疗器械目录的产品的临床试验，需经国家食品药品监督管理总局审核批准后方可正式启动。

上海市第六人民医院伦理委员会 SHANGHAI SIXTH PEOPLE'S HOSPITAL INDEPENDENT ETHICS COMMITTEE

伦理委员会出席成员名单
ETHICS COMMITTEE COMPOSITION

审批编号Approval No：2022-023　　审批日期Date of Review：2022-02-24

委员会成员姓名和职称<br>Member<br>Name and Title		职业<br>Occupation<br>(position)	性别<br>Male/Female	工作单位<br>Working Place
姓名	职称			
贾伟平<br>Jia Wei Ping	教授<br>Professor	医生<br>Doctor	女<br>female	上海市第六人民医院<br>Shanghai Sixth People's Hospital
曾炳芳<br>Zeng Bing Fang	教授<br>Professor	医生<br>Doctor	男<br>male	上海市第六人民医院<br>Shanghai Sixth People's Hospital
郭澄<br>Guo Cheng	教授<br>Professor	药师<br>Pharmacist	男<br>male	上海市第六人民医院<br>Shanghai Sixth People's Hospital
包玉倩<br>Bao Yu Qian	教授<br>Professor	医生<br>Doctor	女<br>female	上海市第六人民医院<br>Shanghai Sixth People's Hospital
周红玲<br>Zhou Hong Ling	研究员<br>Researcher	研究员<br>Researcher	女<br>female	上海空间推进研究所<br>Shanghai Institute of Spacecraft Propulsion
沈成良<br>Shen Cheng Liang	律师<br>Lawyer	律师<br>Lawyer	男<br>male	上海康正律师事务所<br>Shanghai Kangzheng Law Firm
严诚忠<br>Yan Cheng Zhong	教授<br>Professor	教师<br>Teacher	男<br>male	东华大学<br>DongHua University
胡承<br>Hu Cheng	研究员<br>Researcher	研究员<br>Researcher	男<br>male	上海市第六人民医院<br>Shanghai Sixth People's Hospital
沈赞<br>Shen Zan	教授<br>Professor	医生<br>Doctor	男<br>male	上海市第六人民医院<br>Shanghai Sixth People's Hospital
任涛<br>Ren Tao	教授<br>Professor	医生<br>Doctor	男<br>male	上海市第六人民医院<br>Shanghai Sixth People's Hospital
沈成兴<br>Shen Cheng Xing	教授<br>Professor	医生<br>Doctor	男<br>male	上海市第六人民医院<br>Shanghai Sixth People's Hospital
石文静<br>Shi Wen Jing	教授<br>Professor	医生<br>Doctor	女<br>female	上海市第六人民医院<br>Shanghai Sixth People's Hospital
